# Supplementary material for: Maturation process and international accreditation of trauma system in a rapidly developing country
Source: PLoS One. 2020 Dec 10;15(12):e0243658. doi: 10.1371/journal.pone.0243658 (PMC7728290; doi:10.1371/journal.pone.0243658)
Supplement: Attachment — Submitted filename: reply to comments.docx [file pone.0243658.s001.docx]

Journal Requirements:

When submitting your revision, we need you to address these additional requirements.

1. Please ensure that your manuscript meets PLOS ONE's style requirements, including those for file naming. The PLOS ONE style templates can be found at

https://journals.plos.org/plosone/s/file?id=wjVg/PLOSOne_formatting_sample_main_body.pdf and

https://journals.plos.org/plosone/s/file?id=ba62/PLOSOne_formatting_sample_title_authors_affiliations.pdf

2. Your ethics statement should only appear in the Methods section of your manuscript. If your ethics statement is written in any section besides the Methods, please move it to the Methods section and delete it from any other section. Please ensure that your ethics statement is included in your manuscript, as the ethics statement entered into the online submission form will not be published alongside your manuscript.

3. Thank you for including in your ethics statement:

"This observational retrospective 315 study received an expedited review and was
316 approved by the Institutional Review Board (HMC IRB# MRC-01-20-103).".

i) Please amend your current ethics statement to include the full name of the ethics committee/institutional review board(s) that approved your specific study.

ii) Once you have amended this/these statement(s) in the Methods section of the manuscript, please add the same text to the “Ethics Statement” field of the submission form (via “Edit Submission”).

For additional information about PLOS ONE ethical requirements for human subjects research, please refer to http://journals.plos.org/plosone/s/submission-guidelines#loc-human-subjects-research

Reply: done

**Comments to the Author**

5. Review Comments to the Author

Please use the space provided to explain your answers to the questions above. You may also include additional comments for the author, including concerns about dual publication, research ethics, or publication ethics. (Please upload your review as an attachment if it exceeds 20,000 characters)

Reviewer #1: Thank you for the opportunity to review the article “Maturation Process and International Accreditation of Trauma System in a Rapidly Developing Country”.
I commend the authors on being leaders in improving trauma care in Qatar.
To determine if the positive results are because of the improved trauma system, there needs to be;
1. Specific detail about the trauma system and the changes introduced

Reply: thanks, detailed trauma system changes were given in the introduction and discussion in addition to figure 1. See line 81-101 and 216-226

2. A time series analysis of outcomes. It is not possible to do a pre/post analysis based on accreditation, because many of the changes were made leading up to accreditation – so the conclusions are not valid.
You have the data to do this, and it is possible. I suggest this is conducted and the manuscript revised and resubmitted.

Reply: time series analysis done and shown in figure 2

I also have some minor comments around the abstract
The background in the abstract is a hypothesis, there needs to be a little bit of introduction / need for the paper provided – for example, some detail about the changes that were introduced as part of the trauma system

Reply: done in line 30-33

Methods: what type of analyses were performed?

Reply: analyses methods added

In the conclusion you have introduced new results.

Reply: conclusion revised.

Reviewer #2: Thank you for your submission which sought to determine the effects of trauma system maturation to Level 1 trauma care on the outcomes of trauma patients in a rapidly developing country (Qatar).

Abstract: Appropriate representation of the manuscript.

Introduction: Provides thorough information as to the reason for the study.

Patients and methods:
Please add appropriate reference to AIS coding section.

Reply: done , ref 7 and 8 added

It is difficult to see the split of injury severity within the data. The provision of ISS grouping i.e. <12, 12-15, >15 or similar would provide better understanding of the nuances of care for injured groups and also assist in the understanding of access to time critical intervention. An example of data that requires greater understanding is the ED LOS times. 405 mins is significant for those with ISS >15, but not so with those <15.

Reply: new table (3) added with the breakdown of injury severity , GCS and ED time

Results: Appropriately described.

Discussion: See comments

Comments:
The paragraph on education either needs to be related to the study data of removed as it does not currently add value to the discussion

Reply: education paragraph revised.

This is an important body of work and I would like to commend you on your commitment to improving the care provided to injured people and their families. Development and maturation of systems of care are never easy, particularly in developing countries.

Whilst I am enthusiastic about the results here, I do feel that further breakdown and analysis of data is required (as suggested above). I look forward to future developments.

Reply: further analyses done and shown in table 3 and figure 2
